# Genome-Wide Analysis and Functional Characterization of Pyruvate Kinase (PK) Gene Family Modulating Rice Yield and Quality

**DOI:** 10.3390/ijms232315357

**Published:** 2022-12-05

**Authors:** Nannan Dong, Luna Chen, Shakeel Ahmad, Yicong Cai, Yingqing Duan, Xinwei Li, Yongqiang Liu, Guiai Jiao, Lihong Xie, Shikai Hu, Zhonghua Sheng, Gaoneng Shao, Ling Wang, Shaoqing Tang, Xiangjin Wei, Peisong Hu

**Affiliations:** State Key Laboratory of Rice Biology, China National Center for Rice Improvement, China National Rice Research Institute, Hangzhou 311400, China

**Keywords:** pyruvate kinase, yield, quality, rice, gene family

## Abstract

Pyruvate kinase (PK) is one of the three rate-limiting enzymes of glycolysis, and it plays a pivotal role in energy metabolism. In this study, we have identified 10 PK genes from the rice genome. Initially, these genes were divided into two categories: cytoplasmic pyruvate kinase (PKc) and plastid pyruvate kinase (PKp). Then, an expression analysis revealed that *OsPK1*, *OsPK3*, *OsPK4*, *OsPK6*, and *OsPK9* were highly expressed in grains. Moreover, PKs can form heteropolymers. In addition, it was found that ABA significantly regulates the expression of PK genes (*OsPK1*, *OsPK4*, *OsPK9*, and *OsPK10*) in rice. Intriguingly, all the genes were found to be substantially involved in the regulation of rice grain quality and yield. For example, the disruption of *OsPK3*, *OsPK5*, *OsPK7*, *OsPK8*, and *OsPK10* and *OsPK4*, *OsPK5*, *OsPK6*, and *OsPK10* decreased the 1000-grain weight and the seed setting rate, respectively. Further, the disruption of *OsPK4*, *OsPK6*, *OsPK8*, and *OsPK10* through the CRISPR/Cas9 system showed an increase in the content of total starch and a decrease in protein content compared to the WT. Similarly, manipulations of the *OsPK4*, *OsPK8*, and *OsPK10* genes increased the amylose content. Meanwhile, the grains of all CRISPR mutants and RNAi lines, except *ospk6*, showed a significant increase in the chalkiness rate compared to the wild type. Overall, this study characterizes the functions of all the genes of the PK gene family and shows their untapped potential to improve rice yield and quality traits.

## 1. Introduction

Pyruvate kinase (PK) is an enzyme that catalyzes the last step of glycolysis, thereby generating pyruvate and adenosine triphosphate (ATP) by transferring the phosphate group of phosphoenolpyruvates (PEP) to adenosine diphosphate (ADP) in an irreversible reaction [1,2,3,4]. Being one of the three key rate-limiting enzymes in the glycolysis process, PK plays a key role in the pathway starting from glucose to the pro-duction of pyruvate [5]. Pyruvate, the product of the glycolysis pathway, also participates in the regulation of a number of different metabolic pathways [6]. For example, it can produce lactic acid and ethanol under anaerobic conditions and can undergo a decarboxylation reaction under aerobic conditions to produce acetyl-CoA. Then, acetyl-CoA participates not only in the tricarboxylic acid cycle but also in the production of fatty acids and other metabolites [7]. Hence, it shows that PK plays an important role in the energy metabolism of the entire cell [8].

Previously, it has been reported that PK has different isoforms in different organisms. For instance, it has one isoform in lower eukaryotes and bacteria and two (pyruvate kinase F (PykF) and pyruvate kinase A (PykA)) in *E*. *coli* [9,10,11,12,13]. In vertebrates, there are four isoenzymes, including M1, M2, L, and R, that have been found in skeletal muscle, kidney, liver, and red blood cells, respectively [14]. In plants, it has two isozyme forms: (1) cytoplasmic pyruvate kinase (PKc) and (2) plastid pyruvate kinase (PKp). However, the numbers and structures of PK subunits appear to be quite diverse in plants. For example, at least 14 genes (10 PKc genes and 4 PKp genes) in *Arabidopsis thaliana* L. [15], 11 genes (6 PKc genes and 5 PKp genes) in potato [5], and 33 genes (19 PKc genes and 14 PKp) in cotton [16] are responsible for encoding putative PK subunits. Generally, PK exists in the form of homotetramers, but it can also exist as monomers, homodimers, heterodimers, heterotetramers, or heterohexamers [17,18,19,20,21,22]. All together, these PK genes encoding different types of isozymes constitute a huge gene family and participate in various physiological and metabolic processes in plants [23,24].

Many studies have reported the function of PK in the glycolysis pathway in different plant species, including rice, Arabidopsis, tobacco, cotton, etc. For instance, in Arabidopsis, PKp can effectively convert sugars into precursors (i.e., pyruvate, etc.) of different anabolic pathways. These precursors are essential for seed germination and reproduction [23]. However, the deletion of the gene encoding the PKpβ1 subunit leads to a decrease in PKp activity and causes a 60% reduction in seed oil contents. In contrast, the overexpression of *PKpβ1* can completely restore the phenotype, while the overexpression of *PKpβ2* can partially restore the phenotype of seed oil content reduction. Therefore, this shows that PK plays an important role in catalyzing the conversion of photosynthetic products into lipids [20]. In addition, it is reported that the expressions of PKc and PKp genes were decreased and increased, respectively, with the onset of lipid biosynthesis, reflecting a shift in the primary carbon flux from cytosolic to plastid metabolism [25]. In tobacco, the absence of PKc genes resulted in a short-rooted and late-flowering phenotype [26]. Similarly, GhPK6 is a PKc that is specifically expressed in cotton fiber and elongates its length significantly [16]. Likewise, PK genes have also been proven to play an important role in rice growth and development. The mutation of *OsPK1* reduces its expression level, which substantially affects not only the synthesis of gibberellin that eventually causes an imbalance between gibberellin and abscisic acid but also the synthesis, transportation, and metabolism of carbohydrates in plants [27]. At the same time, the lower expression of PKc in the mutant *ospk1* led to a decrease in pyruvate content, which ultimately led the plants towards plant dwarfism and panicle enclosure [28]. Similarly, *OsPK2* (*OsPKα1*) plays an important role in the process of starch synthesis, composite granule formation, and grain filling in rice. In this regard, a mutant of the *OsPK2* (*OsPKα1*) gene in rice showed white-belly endosperm with significant defective grain filling compared to its wild type. It also reduced starch contents and significantly changed their physiochemical properties. Nevertheless, the contents of protein and fat were notably increased [29,30]. Furthermore, the significantly reduced expression of *OsPK3* and *OsPK4* in leaves and endosperm seriously affected the grain filling and the accumulation of storage substances in rice [22].

Although some PK genes have been studied in rice, the biochemical characters and functions of the entire PK family in the rice genome are still largely elusive. In this study, we identified and functionally characterized the whole PK gene family and revealed the role of PK in rice growth and development. Moreover, our results also demonstrate that PK plays an important role in rice grain yield and quality. Finally, our research provides novel insights into the molecular basis of the rice PK gene family and expands our understanding to lay a strong theoretical foundation for the improvement of rice yield and quality in the future.

## 2. Results

### 2.1. Genome-Wide Identification of 10 PK Genes in Rice Genome

Accurate identification and uniform nomenclature are critical for future studies of the rice PK gene family. Here, we identified a total of 10 PK genes from the rice genome and named them *OsPK1* to *OsPK10* (Figure S1; Table S1). The lengths of the genomic and coding sequences of the rice PK genes ranged between 3479 bp and 7340 bp, and 1347 bp and 1752 bp, respectively. The lengths of rice PK proteins, their molecular weights, and their isoelectric points ranged between 448 and 583 amino acids, 48.66 kDa and 63.25 kDa, and 5.91 and 9.19, respectively. Similarly, the grand average of hydropathicity (GRAVY), instability index, and aliphatic index of rice PK proteins were recorded from −0.194 to 0.047, 29.58 to 48.51, and 86.67 to 100.09, respectively (Table S1). In addition, it was observed that several amino acid sites showed high conservation in the Pfam:PK and Pfam:PK_C domains (Figure S2). The molecular weight, a basic characteristic of these PK proteins, of PKp was slightly larger than that of PKc. Proteins (OsPK2, OsPK7, and OsPK9) with an instability index greater than 40 were unstable in vitro. Therefore, these parameters are considerably helpful for analyzing the basic biochemical properties of the protein and for subsequent biochemical experiments related to the protein.

**Figure 1:**
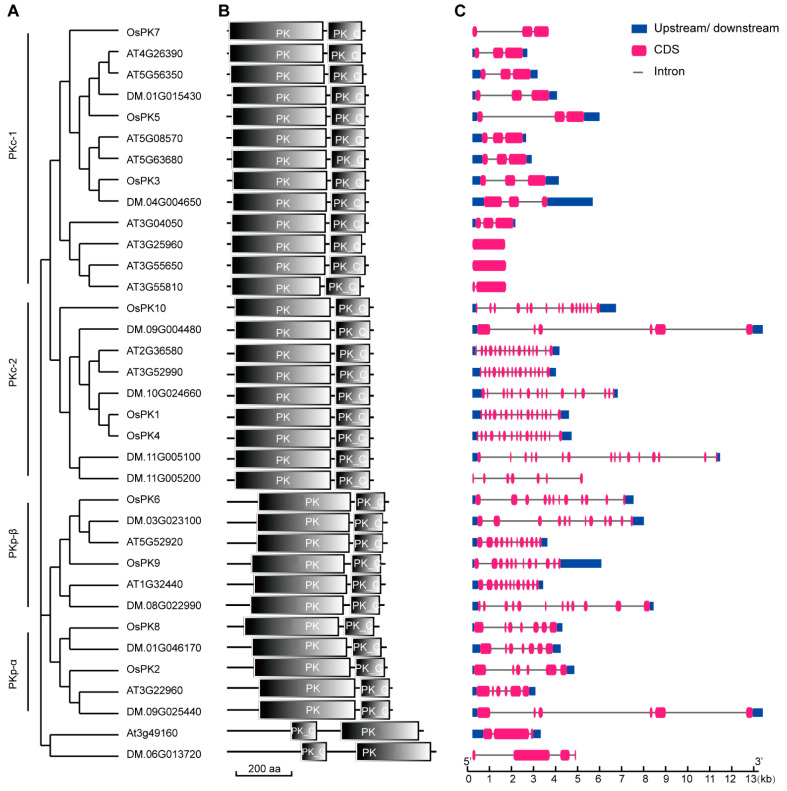
Phylogenetic, motif, and gene structure analyses of PK genes. (**A**) Phylogenetic analysis of PK genes in rice, *Arabidopsis*, and potato. (**B**) Motif analysis of PKs. The scale at the bottom can be used to estimate the lengths of PK proteins. (**C**) Gene structure analysis of PK genes. The pink color represents the exon region, the black line represents the intron region, and the blue color represents the noncoding region. The scale at the bottom can be used to estimate the lengths of PK genes.

Moreover, to explore the evolutionary relationship among the PK proteins of rice, *Arabidopsis*, and potato, a phylogenetic tree was constructed using full-length amino acid sequences of 10, 14, and 11 PK proteins of rice, *Arabidopsis*, and potato, respectively. Among them, rice is not only a monocot but also a food crop, potato is not only a dicot but also a food crop, and Arabidopsis is a monocot model plant. The PK proteins were clustered into two subfamilies, PKc and PKp, and PKc was further clustered into two subunits, PKc-1 and PKc-2. Similarly, PKp was also clustered into two subunits: the α subunit of PKp (PKpα) and the β subunit of PKp (PKpβ) (Figure 1A). In addition, a total of two conserved motifs were identified: Pfam:PK and Pfam:PK_C. Among them, Pfam:PK is the active region of the PK enzyme, whereas Pfam:PK_C is the recognition region of PK (Figure 1B) [31]. To gain more insights into the evolution of genes, the online tool Gene Structure Display Server (GSDS) was used to study the exon–intron structure of the PK genes. The number of introns identified in the PK genes ranged from 0 to 15. However, the structure of PK genes was quite different, especially the genes clustered in the PKc group. Among the two subunits of PKc, PKc-1 showed 0 to 2 introns and PKc-2 exhibited 15 introns. On the other hand, PKp displayed a different gene structure than PKc. In particular, the total number of introns in PKpα was 11, whereas the number of introns in the PKpβ ranged between 2 and 6 (Figure 1C).

### 2.2. Temporal and Spatial Expression Pattern and Subcellular Localization of Rice Pks

**Figure 2:**
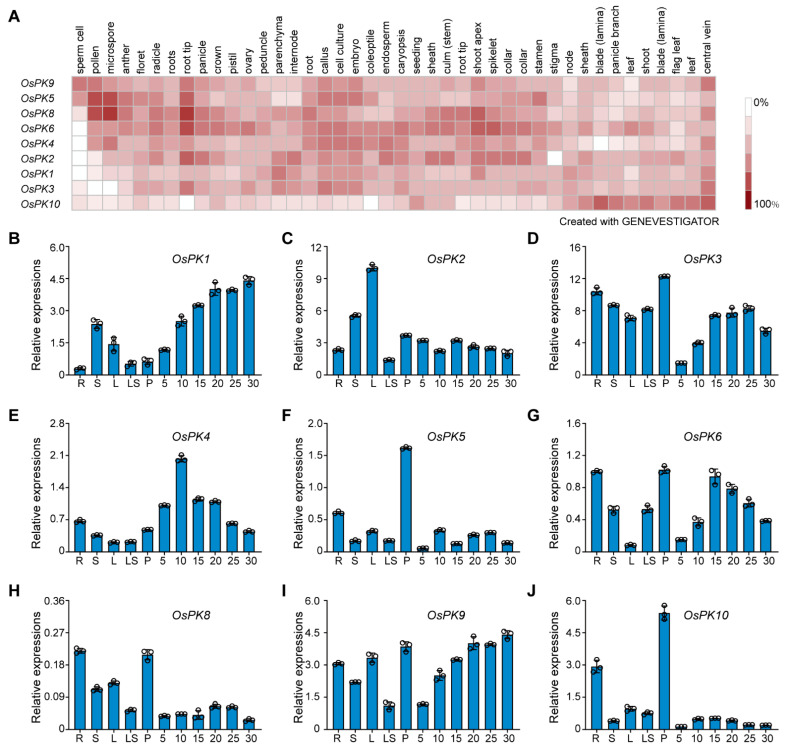
Expression pattern of PK genes in rice. (**A**) Heat map of expression levels of PK genes in rice. A color-based scale from red to white indicates high to low gene expression levels, respectively. (**B**–**J**) Relative expression level of PK genes (*OsPK1*, *OsPK2*, *OsPK3*, *OsPK4*, *OsPK5*, *OsPK6*, *OsPK8*, *OsPK9*, and *OsPK10*) in roots (R), stems (S), leaves (L), leaf sheaths (LS), panicles (P), and seeds at 5, 10, 15, 20, and 25 days after flowering. Error bars indicate the means ± SDs of three individual replicates.

The expression of rice PK genes was analyzed using expression data stored in Genevestigator^®^. The results showed that most of the PK genes were expressed in all tissues (Figure 2A). Hence, real-time quantitative PCR (RT-qPCR) was used to further validate these results and to monitor the precise spatiotemporal expression of PK genes. The results showed that all genes were expressed in various tissues, except *OsPK7*, which could not be detected at any level due to its low expression. Interestingly, during seed development, the expression levels of *OsPK1* and *OsPK9* gradually increased, whereas the expression levels of *OsPK3*, *OsPK4*, *OsPK6*, and *OsPK10* first increased and then decreased. In addition, *OsPK3*, *OsPK5*, *OsPK6*, *OsPK8*, *OsPK9*, and *OsPK10* were highly expressed in the panicles. In roots, *OsPK3*, *OsPK6 OsPK8*, *OsPK9*, and *OsPK10* showed high expression (Figure 2B–J). Finally, the results indicated that PK genes play an important role in the growth and development of rice.

**Figure 3:**
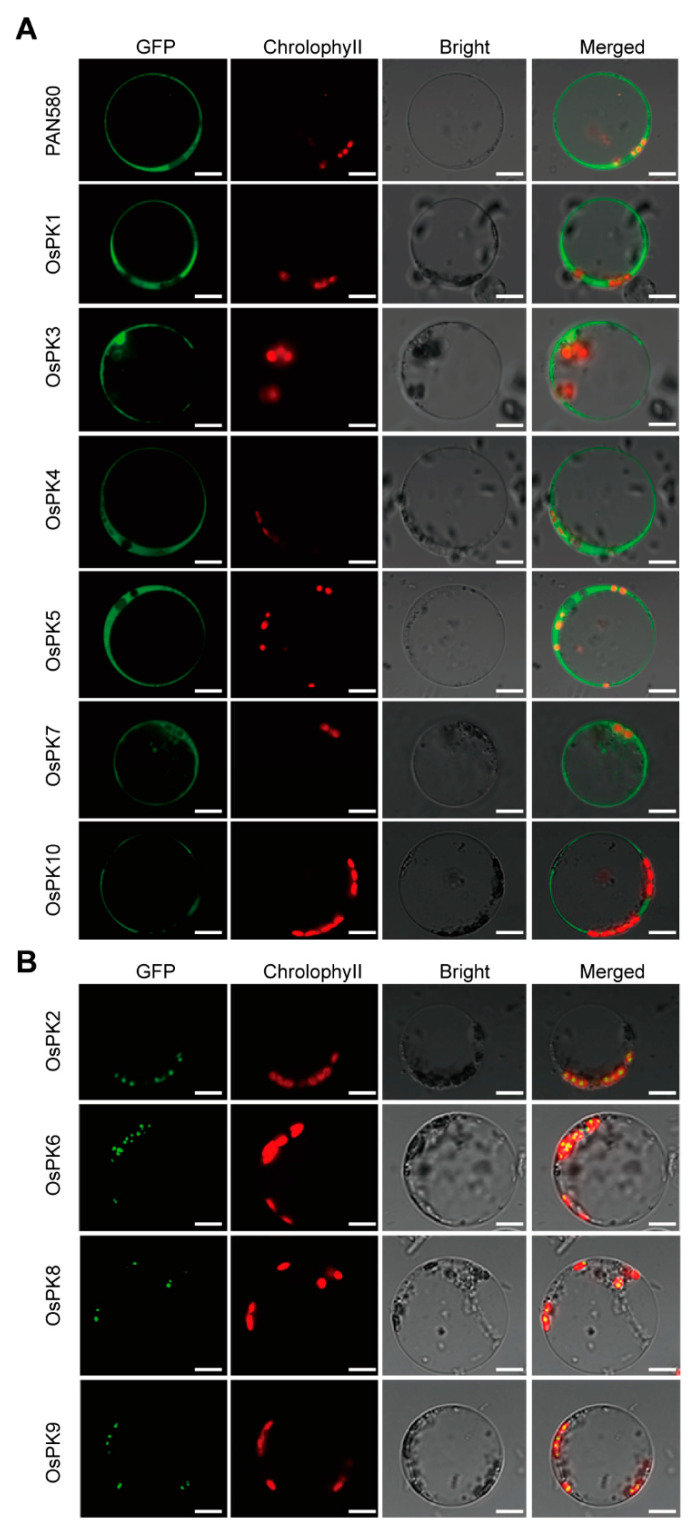
Subcellular localization of PKs in rice. (**A**) Transient expression of GFP, OsPK1-GFP, OsPK3-GFP, OsPK4-GFP, OsPK5-GFP, OsPK7-GFP, and OsPK10-GFP in rice protoplasts. (**B**) Transient expression of OsPK2-GFP, OsPK6-GFP, OsPK8-GFP, and OsPK9-GFP in rice protoplasts. The PKs-GFP fusion proteins were constructed for the subcellular localization assay, and the empty 35S-GFP vector was used as a control. Scale bar = 10 μm.

The members of the PK gene family were divided into two groups (PKc and PKp) in different plant species based on their localization in the cell. Thus, we further verified the subcellular localization of rice PK genes by transforming the PK-GFP fusion proteins into rice protoplasts. The results showed that the green fluorescent signals of PKc (OsPK1, OsPK3, OsPK4, OsPK5, OsPK7, and OsPK10) were located in the cytoplasm, and the green fluorescent signals of PKp (OsPK2, OsPK6, OsPK8, and OsPK9) were located in the chloroplasts. Thus, these findings were found to be consistent with the classification results of the phylogenetic tree. Spectacularly, the signals of PKp were found scattered in the chloroplasts (Figure 3).

### 2.3. PK Proteins of Rice Form Heteropolymers

**Figure 4:**
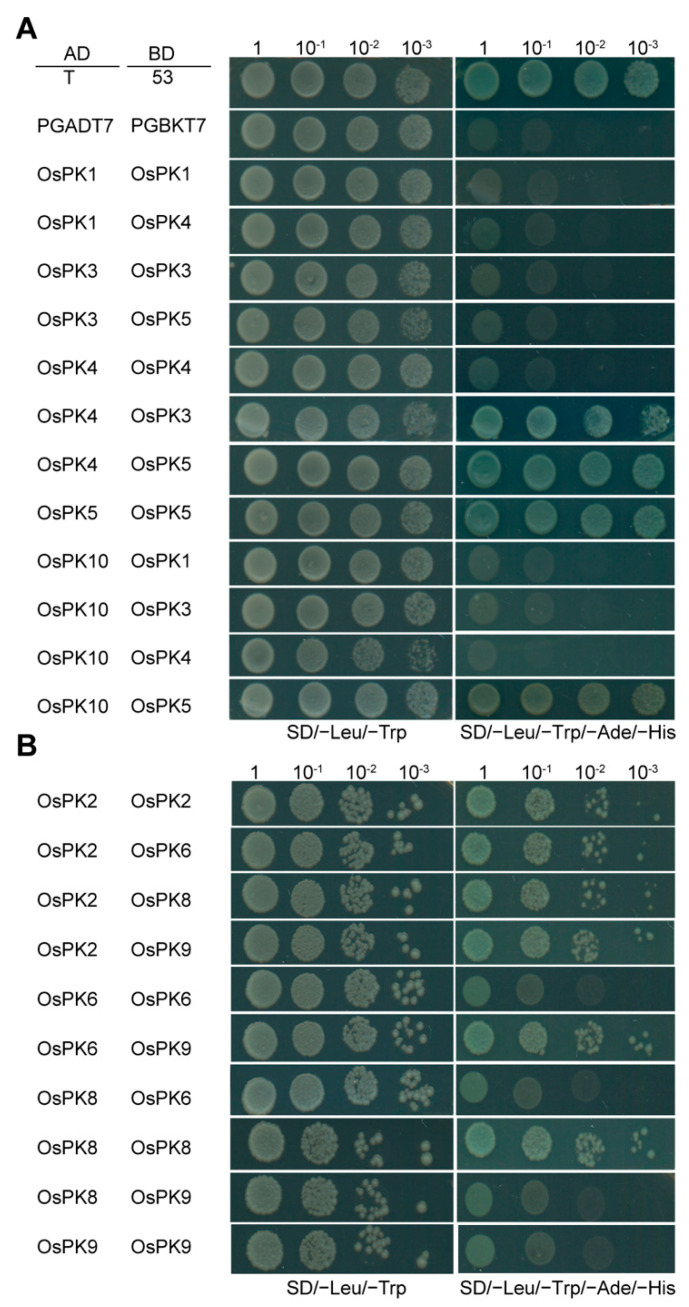
The interactions among PKs in rice. The Y2H assay was used to detect the interactions among the PK proteins and followed the concentration gradient of one tenth on the nonselective pressure medium (SD/−Leu/−Trp) and the selective medium (SD/−Leu/−Trp/−Ade/−His) for dot observation. (**A**) The interactions among PKc. (**B**) The interactions among PKp. The interaction between T (pGADT7-T) and 53 (pGBKT7-53) was used as a positive control. And pGADT7 and pGBKT7 were used as negative controls.

Generally, PK exists in the form of heteropolymers. Hence, the interactions between rice PK proteins have been analyzed using the yeast two-hybrid (Y2H) assay. The results showed that OsPK2, OsPK5, and OsPK8 can interact with themselves. Nevertheless, OsPK3 and OsPK4, OsPK4 and OsPK5, and OsPK5 and OsPK10 can interact with each other. Likewise, OsPK6 and OsPK2, OsPK2 and OsPK8, OsPK2 and OsPK9, and OsPK9 and OsPK6 also showed interactions with each other. However, no interactions were detected between OsPK1 and OsPK10, OsPK3 and OsPK5, OsPK3 and OsPK10, OsPK4 and OsPK1, OsPK4 and OsPK10, OsPK6 and OsPK8, or OsPK8 and OsPK9 (Figure 4; Figure S3). These results indicate that rice PK proteins can form complex polymers to facilitate each other’s functions in rice.

### 2.4. Promoter Cis-Regulatory Element (CRE) Analysis and the Response to Abscisic Acid of PK Genes in Rice

**Figure 5:**
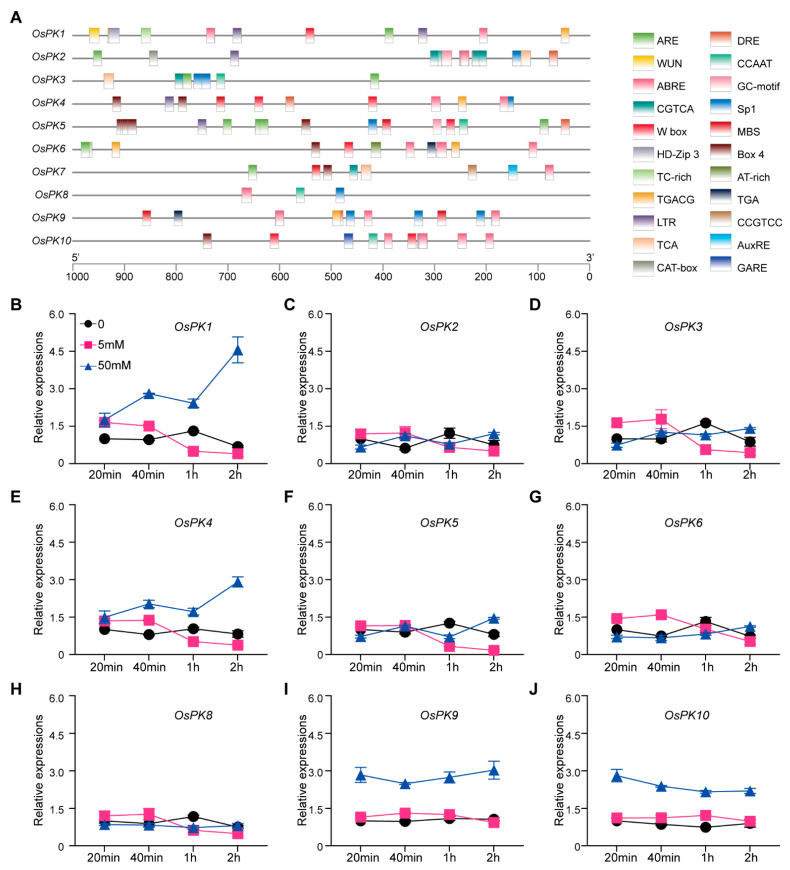
Prediction of promoter element of PK genes in rice and PK gene responses to abscisic acid. (**A**) ARE is a *cis*-acting regulatory element that is essential for anaerobic induction. MBS is an MYB binding site that is involved in drought inducibility. W-box and WUN-motif represent stress-related *cis*-elements. ABRE is an abscisic-acid-responsive element. CGTCA-motif and TGACG-motif are the *cis*-acting regulatory elements involved in MeJA responsiveness. LTR is a *cis*-acting element that is involved in low-temperature responsiveness. Box 4 and Sp 1 are part of a conserved DNA module involved in light responsiveness. AuxRE and TGA are auxin-responsive elements. GARE-motif is a gibberellin-responsive element. (**B**–**J**) The relative expression levels of PK genes (*OsPK1*, *OsPK2*, *OsPK3*, *OsPK4*, *OsPK5*, *OsPK6*, *OsPK8*, *OsPK9*, and *OsPK10*) after treatment with ABA. Black lines represent the group without the addition of abscisic acid. Pink lines represent the treatment group with the addition of 5 mM abscisic acid. Blue lines represent the treatment group with the addition of 50 mM abscisic acid. Error bars indicate the means ± SDs of three individual replicates.

The *cis*-regulatory elements (CREs) in the promoter region of the rice PK genes were analyzed to find upstream regulatory pathways or factors or upstream signals related to family function. The results showed that numerous CREs such as anaerobic response element (ARE), TC-rich, MYB binding site (MBS), wound-responsive element (WUN-motif), W-box, abscisic acid response elements (ABRE), methyl jasmone response elements (CGTCA-motif), gibberellin response element (GARE-motif), etc., were present in the promoter regions of the PK genes. Particularly, ARE *cis*-regulating elements were present in the promoter regions of six PK genes (*OsPK1*, *OsPK2*, *OsPK3*, *OsPK5*, *OsPK6*, and *OsPK7*) that are required for anaerobic induction. Similarly, W-box, CGTCA-motif, MBS, and ABRE CREs were present in the promoter regions of four (*OsPK1*, *OsPK4*, *OsPK5*, and *OsPK6*), seven (*OsPK1*, *OsPK2*, *OsPK3*, *OsPK4*, *OsPK6*, *OsPK7*, and *OsPK9*), four (*OsPK4*, *OsPK5*, *OsPK7*, and *OsPK9*), and eight (*OsPK1*, *OsPK2*, *OsPK4*, *OsPK6*, *OsPK7*, *OsPK8*, *OsPK9*, and *OsPK10*) PK genes, respectively (Figure 5A). The presence of these elements indicates that PK genes may play a role in hormone signal transduction and biotic and abiotic stress responses.

In light of the results of the CRE analysis of eight rice PK genes with ABRE, two-week-old rice seedlings were grown under normal growth conditions and treated with ABA (abscisic acid) to check the expression levels of the rice PK genes. The expression was checked after 20 min, 40 min, 1 h, and 2 h of ABA treatment. Different concentrations of ABA, including 0 mM, 0.5 mM, and 50 mM, were used to check the gene expression. It was recorded that the expression levels of *OsPK1*, *OsPK4*, *OsPK9*, and *OsPK10* under the treatment with 50 mM ABA were significantly higher than those of the control, while the expression of the remaining genes under the treatments with 5 mM or 50 mM ABA had no significant differences compared with the control (Figure 5B–J). These results show that ABA triggers the expression of the *OsPK1*, *OsPK4*, *OsPK9*, and *OsPK10* genes.

### 2.5. Creation of CRISPR Mutants and Rnai Lines of PK Genes in Rice

**Figure 6:**
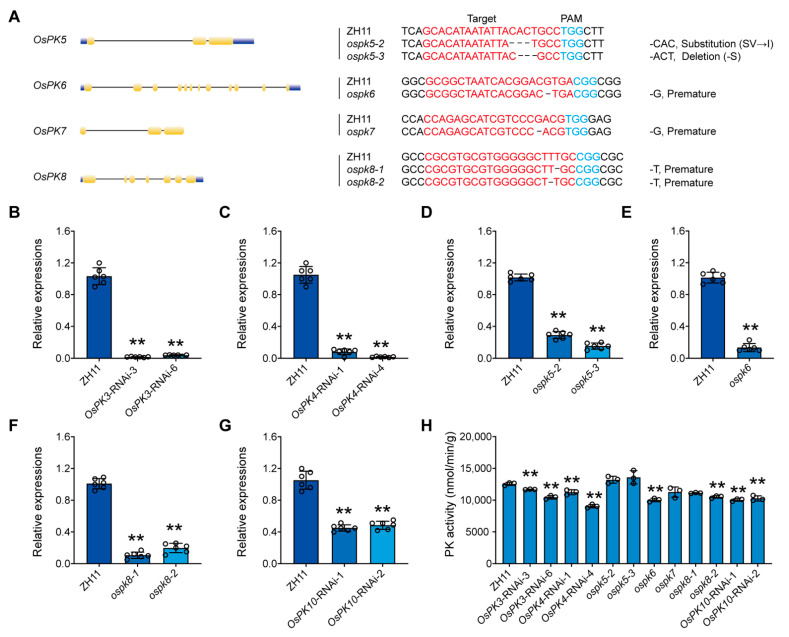
The acquisition of the CRISPR/Cas9-based mutants and RNAi lines of PK genes in rice. (**A**) The structure, PAM sequence, and editing of *OsPK5*, *OsPK6*, *OsPK7*, and *OsPK8*. (**B**–**G**) The relative expression of *OsPK3, OsPK4, OsPK5, OsPK6, OsPK8*, and *OsPK10* in the corresponding lines. (**H**) Pyruvate kinase activity of grains ten days after flowering. Error bars indicate the means ± SDs of six individual replicates. Asterisks indicate statistical significance between the WT and the CRISPR mutants or RNAi lines, as determined by Student’s *t*-test (** *p* < 0.01).

Both RNAi and CRISPR/Cas9 techniques were used to obtain mutants for studying the biological functions of PK genes, which also validated each the other results. In this regard, *OsPK3*-RNAi-3, *OsPK3*-RNAi-6, *OsPK4*-RNAi-1, *OsPK4*-RNAi-4, *OsPK10*-RNAi-1, and *OsPK10*-RNAi-2 were generated using the RNAi technique to knock down the expression of the respective genes in the rice cultivar ZH11. The results showed that the *OsPK3*-RNAi-3, *OsPK3*-RNAi-6, *OsPK4*-RNAi-1, *OsPK4*-RNAi-4, *OsPK10*-RNAi-1, and *OsPK10*-RNAi-2 lines significantly decreased the expression of their respective genes compared to the wild type (WT). Similarly, *ospk5-2*, *ospk5-3*, *ospk6*, *ospk7*, *ospk8-1*, and *ospk8-2* were generated using a CRISPR/Cas9-based gene editing system in same rice cultivar. Several CRISPR-based mutant plants were obtained through the CRISPR/Cas9 system, whereas one or two lines of each gene were studied further. For example, it was observed that the *ospk5-2* mutant line exhibited a three-nucleotide deletion, which resulted in the replacement of serine and valine with isoleucine. Similarly, the *ospk5-3* line also showed a three-nucleotide deletion, which resulted in the loss of serine. Likewise, a one-nucleotide base-pair deletion was found in the *ospk6*, *ospk7*, *ospk8-1*, and *ospk8-2* lines, which caused the early termination of the translation process (Figure 6A). These results showed that both the CRISPR/Cas9 and RNAi systems fully knocked out or decreased the expression of PK genes, respectively. Consequently, the activity of PK was significantly decreased in the fresh seeds of both the CRISPR and RNAi lines (Figure 6B–H). Besides all these results, *OsPK1* and *OsPK2* genes were not targeted via the CRISPR/Cas9 system because they have already been studied, and their results are discussed below. However, we remained unsuccessful in obtaining CRISPR mutants of the *OsPK9* gene.

### 2.6. PK Modulates the Formation of Rice Yield and Quality

**Figure 7:**
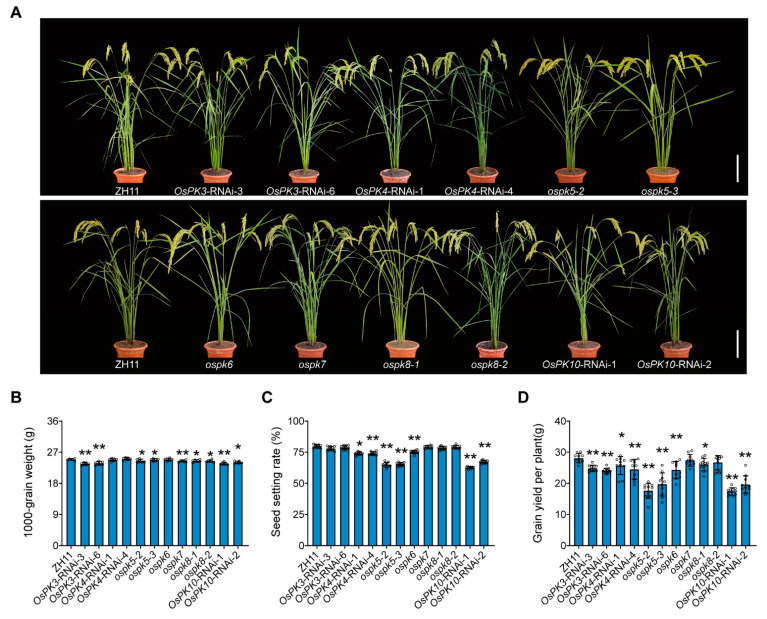
Yield-related traits of ZH11, CRISPR/Cas9-based mutants, and RNAi-based material of PK genes in rice. (**A**) The mature plants of ZH11, *OsPK3*-RNAi-3, *OsPK3*-RNAi-6, *OsPK4*-RNAi-1, *OsPK4*-RNAi-4, *ospk5-2*, *ospk5-3*, *ospk6*, *ospk7*, *ospk8-1*, *ospk8-2*, *OsPK10*-RNAi-1, and *OsPK10*-RNAi-2. Scale bars = 20 cm. (**B**–**D**) The 1000-grain weight, seed setting rate, and grain yield per plant of ZH11, the CRISPR/Cas9-based mutants, and RNAi material. *n* = 6 (B), *n* = 10 (C), *n* = 10 (D). Error bars indicate the means ± SDs. Asterisks indicate statistical significance between the WT and the CRISPR/Cas9-based mutants or RNAi-based materials, as determined by Student’s *t*-test (* *p <* 0.05; ** *p <* 0.01).

The yield traits of the mature plant of *cv*. ZH11 (WT), CRISPR mutants, and RNAi lines were measured to determine the contribution of PK genes in rice yield. The results of *OsPK3*-RNAi-3 and *OsPK3*-RNAi-6 showed that the grain thickness, 1000-grain weight, and grain yield per plant were significantly reduced compared to the WT, whereas the down regulation of the *OsPK4* gene in the *OsPK4*-RNAi-1 and *OsPK4*-RNAi-4 lines only affected the seed setting rate and grain yield per plant compared to the WT. Interestingly, the silencing of *OsPK10* affected not only the grain thickness, 1000-grain weight, seed setting rate, and grain yield per plant but also the grain width in the *OsPK10*-RNAi-1 and *OsPK10*-RNAi-2 lines compared to the WT. On the other hand, CRISPR mutants also exhibited similar results. For instance, the 1000-grain weight, seed setting rate, and grain yield per plant were significantly decreased in the knockout mutants *ospk5-2* and *ospk5-3* compared to the WT. Similarly, *ospk6* merely showed declines in the seed setting rate and grain yield per plant compared to the WT. Moreover, *ospk7* only showed a decrease in the 1000-grain weight compared to the WT. In the cases of the *ospk8-1* and *ospk8-2* CRISPR mutants, the grain width, grain thickness, 1000-grain weight, and grain yield per plant were significantly reduced compared to the WT (Figure 7A–D; Figure S4).

**Figure 8:**
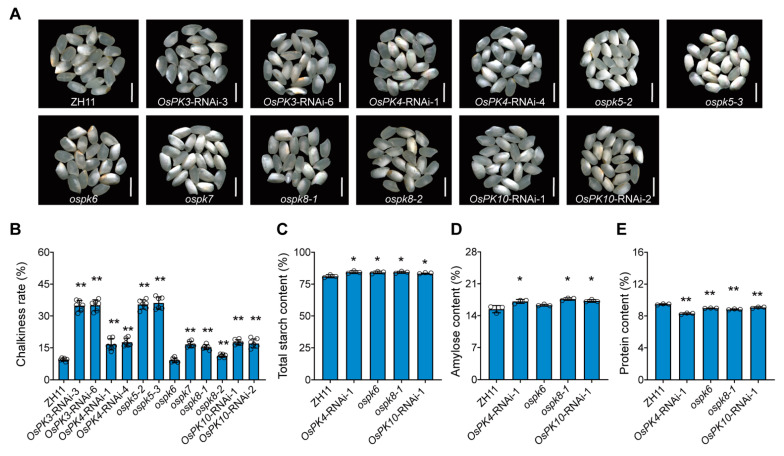
Grain quality of ZH11, CRISPR/Cas9-based mutants, and RNAi-based material with respect to PK genes in rice. (**A**) Phenotypes of seeds of ZH11, *OsPK3*-RNAi-3, *OsPK3*-RNAi-6, *OsPK4*-RNAi-1, *OsPK4*-RNAi-4, *ospk5-2*, *ospk5-3*, *ospk6*, *ospk7*, *ospk8-1*, *ospk8-2*, *OsPK10*-RNAi-1, and *OsPK10*-RNAi-2. Scale bars = 5 mm. (**B**) Chalkiness rate of the WT, mutant, and RNAi material seeds. Error bars indicate the means ± SDs of six biological replicates. (**C**–**E**) Total starch contents, amylose contents, and protein contents of WT, mutant, and RNAi line seeds. Error bars indicate the means ± SDs of three individual replicates. Asterisks indicate statistical significance between the WT and the mutants and RNAi material seeds, as determined by Student’s *t*-test (*p < 0.05; ** *p* < 0.01).

In addition, the rice grain quality traits of the CRISPR mutants and RNAi lines were also compared with the WT. It was observed that the grains of all CRISPR mutants and RNAi lines, except *ospk6*, showed significant increases in the chalkiness rate compared to the WT (Figure 8A,B). Further, the contents of stored substances in the grains were measured as well. In this regard, *OsPK4*-RNAi-1, *ospk6*, *ospk8-1*, and *OsPK10*-RNAi-1 showed increases in the contents of total starch and decreases in the protein content compared to the WT (Figure 8C,E). Additionally, *OsPK3*-RNAi-3, *OsPK4*-RNAi-1, *ospk5*, *ospk8-1*, and *OsPK10*-RNAi-1 showed increases in the contents of amylose compared to the WT (Figure 8D; Figure S4). Based on these results, it is concluded that *OsPK4*, *OsPK6*, *OsPK8*, and *OsPK10* are positive regulators of the protein content but negative regulators of the total starch. Likewise, *OsPK3*, *OsPK4*, *OsPK5*, *OsPK8*, and *OsPK10* are negative regulators of the amylose content.

## 3. Discussion

The overall study of gene families can provide effective theoretical support to study the functions of genes. In this study, 10, 14, and 11 PK genes were identified from the whole genomes of rice, Arabidopsis, and potato, respectively (Figure 1A). There are at least 19 PKc genes and 14 PKp genes in cotton [16]. In parallel, the results of the current evolutionary study, performed via a phylogenetic analysis, clearly showed the two groups (PKc and PKp) of the PK gene family, which are consistent with the classification results of Arabidopsis and potato (Figure 1A). In addition, a protein conservative motif analysis showed that most of the PK proteins had two conservative motifs, Pfam:PK and Pfam:PK_C. The functions of the PK proteins in various species are relatively conserved, and these two motifs contribute to their recognition and functional activities (Figure 1B). Though the genetic structures of the PK genes are very different, the number of introns in each small branch is similar. This indicates that the PK genes, during their evolutionary process, have changed the numbers and the lengths of introns to adapt to the environment but retained their conserved domains, which enabled the genes to perform their functions stably (Figure 1C). Therefore, PK exists in most of the organisms as a homotetramer, whereas it can also be present in the form of a monomer, homodimer, heterodimer, heterotetramer, or heterohexamer and can also exist in the form of a homodecamer [1,32,33]. However, the subunit composition of the PK complex depends on the species, tissue, and subcellular localization [34]. For example, in Arabidopsis, PKp exists in the form of 4α4β_1_ [22], whereas in current study it was found that the PK genes exist in the form of heteropolymers in rice (Figure 4 and Figure S3). In plants, the spatiotemporal expression pattern of genes is critically linked with their function, and it can provide important information for studying gene functions. PK genes are all constitutively expressed in various tissues, but their functions are different (Figure 2). For example, *OsPK2* affects the accumulation of starch in rice seed endosperm [28,29], while *OsPK3* and *OsPK4* affect the transportation of sucrose and the filling process of seeds [22]. The most direct way to study gene function is to obtain loss-of-function or gain-of-function mutants of the genes. Therefore, CRISPR/Cas9-based mutants and RNAi lines of PK genes were generated that showed the phenotype of increased chalkiness and decreased grain yield per plant (Figure 7 and Figure 8). Similarly, it has been reported that *ospk2*, *ospk3*, and *ospk4* possess a chalky phenotype [22,28,29], which indicates that PK proteins play an important role in the process of seed filling. The chalky phenotype of rice seeds has a great impact on rice seed production. Previously, several reports have been published and found that loss-of-function mutants such as *osgbp*, *osbt1*, *flo16*, and *flo18* produced a chalky phenotype that impacted the rice yield [35,36,37,38]. In fact, there are many reasons behind the development of chalkiness in rice grains. One of those reasons is a reduction in acetyl-CoA, a substrate of the tricarboxylic acid cycle, which leads to a disturbance in energy metabolism in mitochondria that leads to the changes in starch and protein content in grains and ultimately give rise to the formation of a chalky phenotype and low yields [39,40]. Similarly, we speculate that the chalkiness in the rice grains of our CRISPR/Cas9-based mutants or RNAi lines could be due to the lack of PK, leading to a decrease in pyruvate content. Therefore, the content of acetyl-CoA produced by the decarboxylation of pyruvate under the action of the pyruvate dehydrogenase complex may be reduced and ultimately lead to the chalky grains.

Additionally, it was reported that ABA regulates many important processes in plant life [41]. It plays an important role in the responses of plants to salt, drought, cold, and oxidation stresses [42,43,44]. Hence, our research shows that *OsPK1*, *OsPK4*, *OsPK9*, and *OsPK10* are induced by ABA. Therefore, we postulate that these four genes may play an important role in plants in the response to abiotic stresses (Figure 5). Similarly, it was also described in previous reports that the decreased expression of *OsPK1* and *OsPK10* leads to a change in the balance between ABA and gibberellins in rice, which is consistent with the results of our ABA treatment experiments [3,27]. Intriguingly, the *OsPK4*-RNAi-1, *OsPK4*-RNAi-4, *OsPK10*-RNAi-1, and *OsPK10*-RNAi-2 lines showed a chalky phenotype (Figure 8A), which triggered speculation that WT plants transmit signals to PK in response to ABA, which activates the activity of pyruvate kinase to ensure normal energy transportation, while the plants that lack the activity of PK block the energy supply (Figure 6), resulting in a phenotype with a low germination rate, a low seedling rate (Figure S5), high chalkiness, and a low plant yield (Figure 7 and Figure 8). Therefore, we floated an idea to study the effect of PK on rice grain quality and yield. Moreover, chalkiness is not only controlled by genes but can also easily be affected by high-temperature conditions [45,46,47,48]. Therefore, in this study, the CRISPR mutants, RNAi lines, and WT plants were grown in the field in high-temperature conditions of 36/27 °C, day/night, and the differences in the chalkiness and 1000-grain weight were calculated, which could be due to these severe environmental conditions. Hence, it was easy to correlate the high temperature and chalkiness. Thus, we speculate that the enzymatic activity of PK can be affected at high temperatures that may disrupt energy metabolism and can eventually lead to more severe chalkiness. Because chalkiness is a complex trait, previous studies have also identified many quantitative trait loci (QTLs) that control chalkiness in rice at high temperatures [49,50,51,52]. In this regard, three QTLs controlling chalkiness have been detected in japonica rice varieties under high-temperature stress [53]. Thus, all the correlations and findings of the present study suggest that PK plays an important role in the growth and development of rice. It may also play a critical role in plant homeostasis by participating in energy metabolism when plants are exposed to stress such as high temperatures.

## 4. Materials and Methods

### 4.1. Plant Materials and Growth Conditions

The *japonica* rice cultivar Zhonghua 11 (*japonica cv*. ZH11), obtained from China National Rice Research Institute, was used in this study. Mutant plants were generated using different genetic engineering tools, including CRISPR/Cas9 and RNAi systems. For example, *OsPK3*-RNAi-3, *OsPK3*-RNAi-6, *OsPK4*-RNAi-1, *OsPK4*-RNAi-4, *OsPK10*-RNAi-1, and *OsPK10*-RNAi-2 were obtained using an RNAi system in *japonica* cv. ZH11. The CRISPR mutants *ospk5-2*, *ospk5-3*, *ospk6*, *ospk7*, *ospk8-1*, and *ospk8-2* of *japonica* cv. ZH11 were generated via a CRISPR/Cas9 system. All plants mentioned in this study were grown under normal conditions in a field or in a greenhouse at China National Rice Research Institute, Hangzhou, China. Meanwhile, standard agronomic practices were followed throughout their cultivation process.

### 4.2. Identification of PKs

The amino acid sequences, genomic DNA sequences, and coding sequences (CDSs) of the members of PK gene family in *japonica* rice, Arabidopsis, and potato were obtained from the National Center for Biotechnology Information (NCBI, https://www.ncbi.nlm.nih.gov), The Arabidopsis Information Resource (https://www.arabidopsis.org/index.jsp), and the Spud Database (http://solanaceae.plantbiology.msu.edu/index.shtml), accessed on 1 March 2021, respectively. However, the locus name; CDS coordinates (5’-3’); length of genomic DNA, CDS, and amino acids; and molecular weight were collected from the website https://www.ricedata.cn/ of the China Rice Data Center, accessed on 3 March 2021. Their isoelectric points, GRAVY values, instability indices, and aliphatic indices were obtained from the website https://web.expasy.org/protparam/ of Expasy, accessed on 3 March 2021.

### 4.3. Multiple Sequence Alignment, Phylogenetic Analysis, Motifs, and Gene Structure Analysis

The results of multiple sequence alignment were obtained from the NCBI website (https://www.ncbi.nlm.nih.gov/tools/cobalt/) using full-length amino acid sequences of 35 PK proteins, accessed on 2 April 2021. Then, MEGA7 software was used for the construction of the unrooted phylogenetic tree via the neighbor-joining method [54]. The consistency of the unrooted phylogenetic tree was validated by setting an ultrafast bootstrap value of 500. The conserved domain analysis was performed using the online SMART tool (http://smart.embl-heidelberg.de/smart/set_mode.cgi?NORMAL=1), accessed on 4 August 2021. For the gene structure analysis, the genomic DNA sequences were uploaded to the Gene Structure Display Server (http://gsds.gao-lab.org/index.php) in FASTA format, accessed on 4 August 2021, and results were obtained.

### 4.4. Expression Pattern of PK Family Genes in Rice

The expression pattern of PK family genes was checked through RT-qPCR. To perform RT-qPCR, total RNA was extracted from different parts of rice plants using the Plant RNA Extraction Kit (Bio Teke, Beijing, China). Samples from roots, stem, leaves, leaf sheaths, panicles, and seeds at 5, 10, 15, 20, 25, and 30 days after flowering were prepared for the total RNA isolation. Then, a ReverTra Ace qPCR RT Kit (Toyobo, Osaka, Japan) was used for first-strand cDNA synthesis. Finally, the PCR reaction was carried out in a light Cycler 480 system (Roche, Basel, Switzerland), and the results were calculated using the 2^−ΔΔCT^ method [55]. The primers used in RT-PCR are listed in Table S2. Moreover, in silico expression data of the PK family genes were obtained from the Affymatrix Rice Genome Array platform (OS_AFFY_RICE-0), and the heat map was made with the software Genevestigator^®^.

### 4.5. Subcellular Localization

The coding sequence of PK genes without termination codons was cloned into the pAN580-GFP vector. All fusion constructs, including OsPK1-GFP, OsPK2-GFP, OsPK3-GFP, OsPK4-GFP, OsPK5-GFP, OsPK6-GFP, OsPK7-GFP, OsPK8-GFP, OsPK9-GFP, and OsPK10-GFP, were transformed into the protoplast of rice. The transformation was conducted as has been described previously [56]. A confocal laser scanning microscope (Karl Zeiss, Jena, Germany) was used for the observation of fluorescent signals by particular proteins. The primers used in the subcellular localization experiment are listed in Table S2.

### 4.6. Yeast Two-Hybrid Assays

To construct the vectors for the Y2H assay, the coding sequences of *OsPK1*, *OsPK2*, *OsPK3*, *OsPK4*, *OsPK5*, *OsPK6*, *OsPK7*, *OsPK8*, *OsPK9*, and *OsPK10* were amplified through PCR and cloned into the pGADT7 prey vector and pGBKT7 bait vector. The vector transformation and selection were followed as written in Clontech’s guidebook. The primers used in the Y2H assays are listed in Table S2.

### 4.7. Identification of Cis-Regulatory Elements in the Promoter Region

The website of Plant CARE (http://bioinformatics.psb.ugent.be/webtools/plantcare/html/, accessed on 3 September 2021), was used for the prediction of *cis*-regulatory elements in the promoter region of every PK gene in rice by uploading the sequence 1 kb upstream of the start codon. The *cis*-regulatory elements identified in the promoters were displayed with the software TBtools.

### 4.8. Identification of the Positions of the Genes on the Chromosome

Map Gene2 Chromosome v2 (MG2C) was used for the identification of the positions of the genes on the chromosome (http://mg2c.iask.in/mg2c_v2.0/, accessed on 23 February 2021).

### 4.9. Measurement of PK Enzyme Activity

The activity of the PK enzyme was measured by following the procedure based on the principle of the coupling reaction of pyruvate and NADH to NAD^+^. In this regard, the crude enzyme solution was extracted from the fresh tissue, and the samples were further processed as per the method described previously [24]. A microplate reader was used (Infinite 200 PRO, TECAN, Männedorf, Switzerland) to measure the change in absorbance at 340 nm of the reaction system to calculate the PK activity.

### 4.10. Determination of Total Starch and Protein Contents in Endosperm

The total starch and amylose contents of mature endosperm were measured using the Megazyme K-TSTA and K-AMYL starch assay kits. The protein contents were measured following the previously described method [57].

### 4.11. Seed Germination Experiment

Fifty healthy seeds of ZH11, CRISPR/Cas9-based mutant, and RNAi lines were placed in petri dishes (9 cm in diameter) with filter paper and 7 mL of distilled water. All petri dishes were incubated in an incubator for 7 days at a constant temperature of 28 °C with a 12 h/12 h light–dark cycle. The seed germination data were collected on a daily basis. In this regard, the radicle length ≥2 mm was set as a standard to count the number of germinated seeds. Seedlings were considered developed when the roots reached the length of the seed and the shoots reached half the length of the seed. The germination rate (GR) was calculated as GR = (total germinated seeds/50) × 100, and the germination index (GI) was calculated as GI = ∑(Gt/t), where Gt is the number of seeds germinated on day t. This study included three replicates.

## 5. Conclusions

In this study, the whole genomic atlas PK gene family was explored in rice. A total of 10 genes were identified from the PK gene family and were further categorized into two groups: PKc and PKp. These genes play important roles in different biological functions that are performed in different plant tissues. Thus, they were found to affect the yield and quality of rice grains by participating in endosperm filling processes. Finally, the current study provides significant insights regarding the function of the PK gene family in rice and opens various novel avenues to accelerate the rice breeding program to achieve global food security.

## Data Availability

The datasets presented in this study can be found in the article and Supplementary Material.

## Supplementary Materials

The following supporting information can be downloaded at: https://www.mdpi.com/article/10.3390/ijms232315357/s1.
