# Supplementary material for: Genome-Wide Analysis and Functional Characterization of Pyruvate Kinase (PK) Gene Family Modulating Rice Yield and Quality
Source: Int J Mol Sci. 2022 Dec 5;23(23):15357. doi: 10.3390/ijms232315357 (PMC9739881; doi:10.3390/ijms232315357)
Supplement: Supplementary file 1 [file ijms-23-15357-s001.zip › ijms-2077178-supplementary.pdf]

Supplementary Material

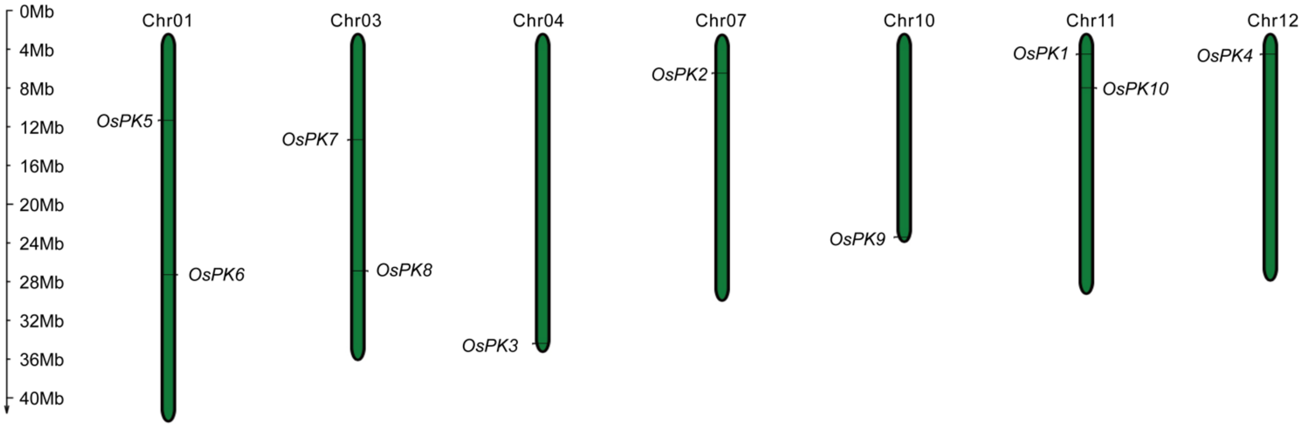

Figure S1. Chromosome localization analysis of PK genes in rice. The chromosome number is displayed at the top of each bar, and the size of the chromosome is indicated by its relative length. The unit on the left scale is Mb, and the short line indicates the approximate location of the PK genes on the corresponding chromosome.

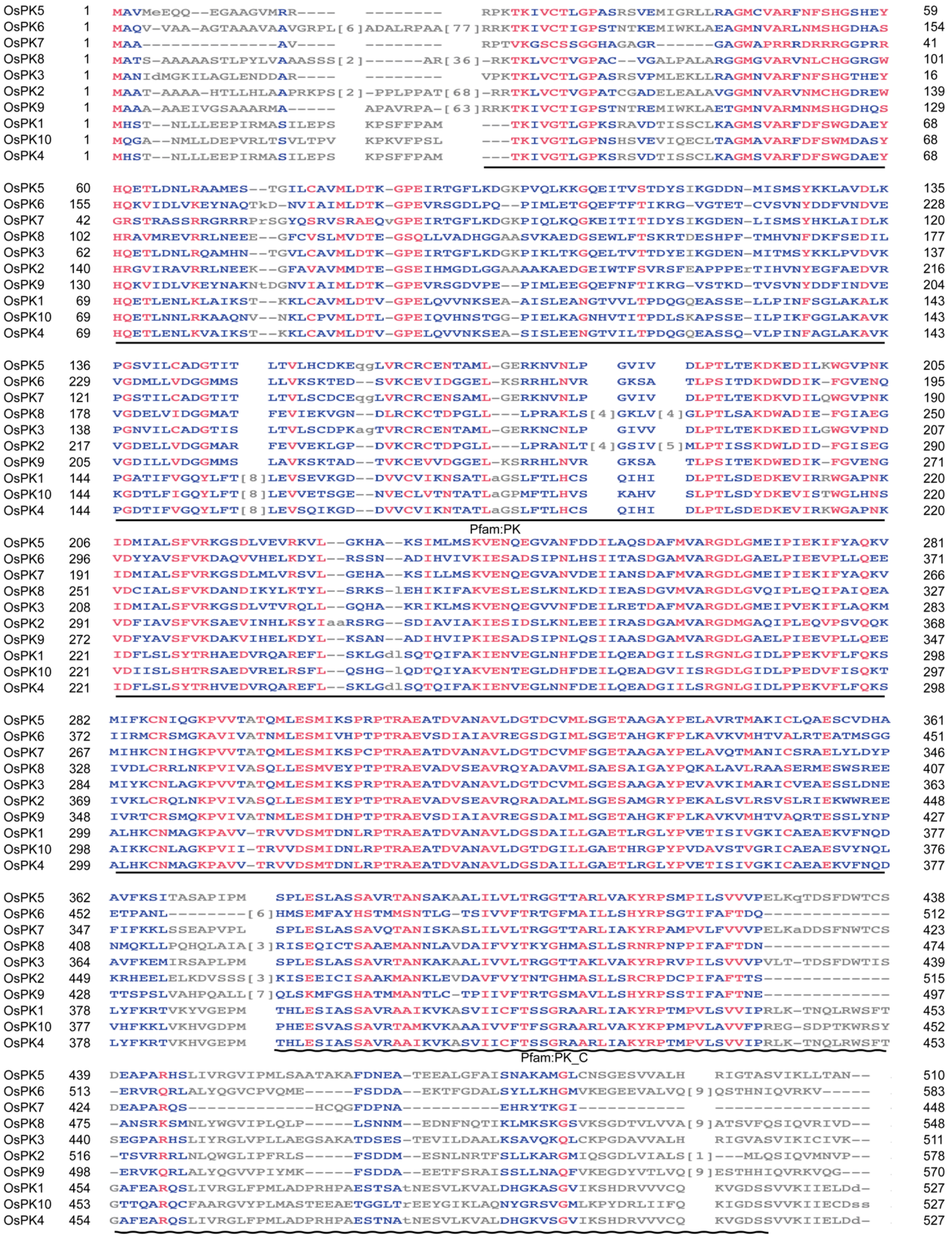

Figure S2. Multiple sequence alignment among PKs in rice. Constraint-based Multiple Alignment Tool was used for the multiple sequence alignment. The red color indicates highly conserved columns and blue indicates less conserved ones. The long black line represents the Pfam:PK domain and the black wavy line represents the Pfam:PK_C domain.

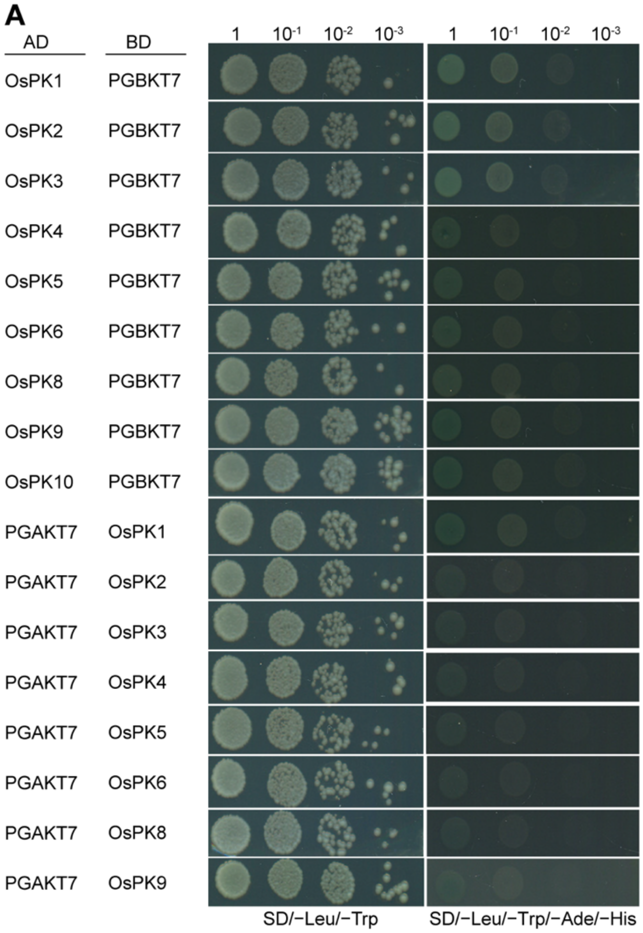

Figure S3. Interaction among PKs in rice. Y2H assay was used to detect interaction among PKs. Follow the concentration gradient of one-tenth on the non-selective pressure medium (SD/−Leu/−Trp) and the selective medium (SD/−Leu/−Trp/−Ade/−His) for dot observation. The interaction between T (pGADT7-T) and 53 (pGBKT7-53) was used as a positive control. And pGADT7, pGBKT7 were used as negative control.

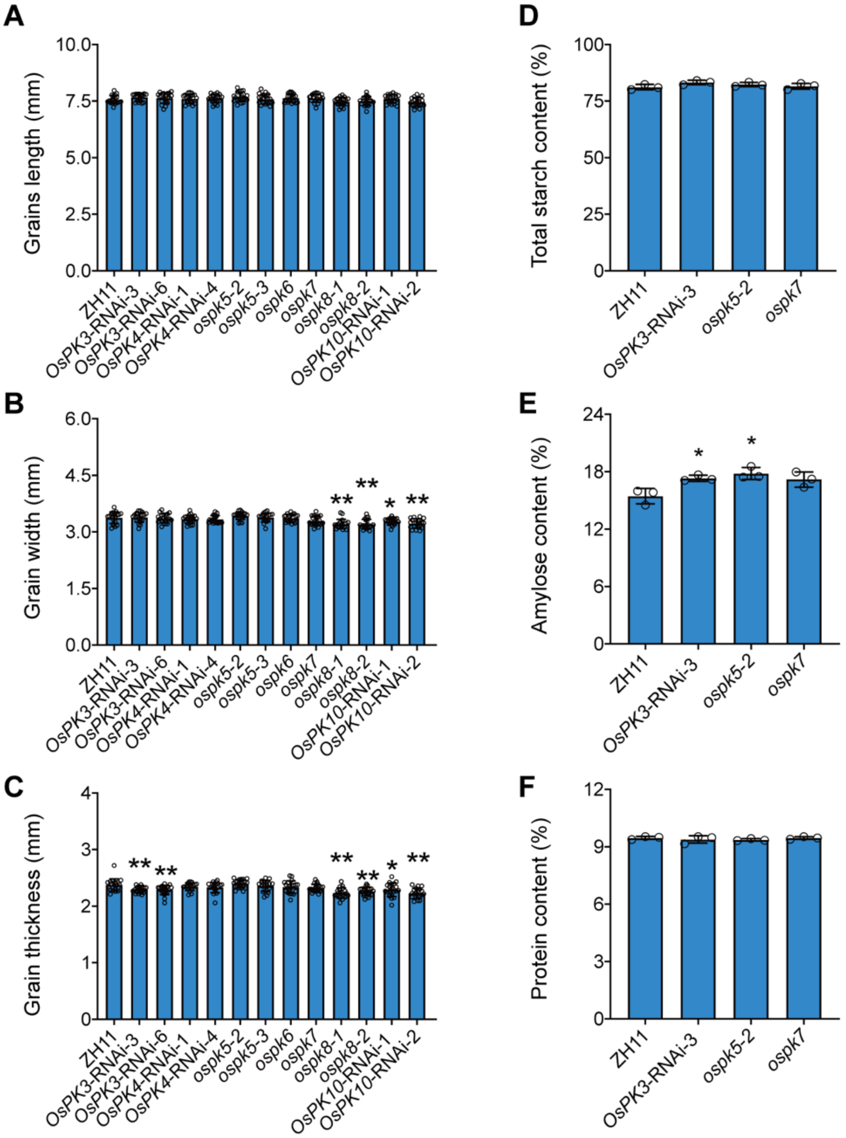

Figure S4. Yield-related traits and grain quality of ZH11, CRISPR/Cas9-based mutants, and RNAi-based material of PK genes in rice. **(A-C)** Grain length, grain width, and grain thickness of ZH11, the CRISPR/Cas9-based mutants, and RNAi material. *n*=20. Error bars indicate the mean ± SD. **(D-F)** Total starch content, amylose content, and protein content of WT and mutants and RNAi lines seeds. Error bars indicate the mean ± SD of three individual replicates. Asterisks indicate statistical significance between the WT and the CRISPR/Cas9-based mutants or RNAi-based materials, as determined by a Student's *t*-test (*$P < 0.05$; **$P < 0.01$).

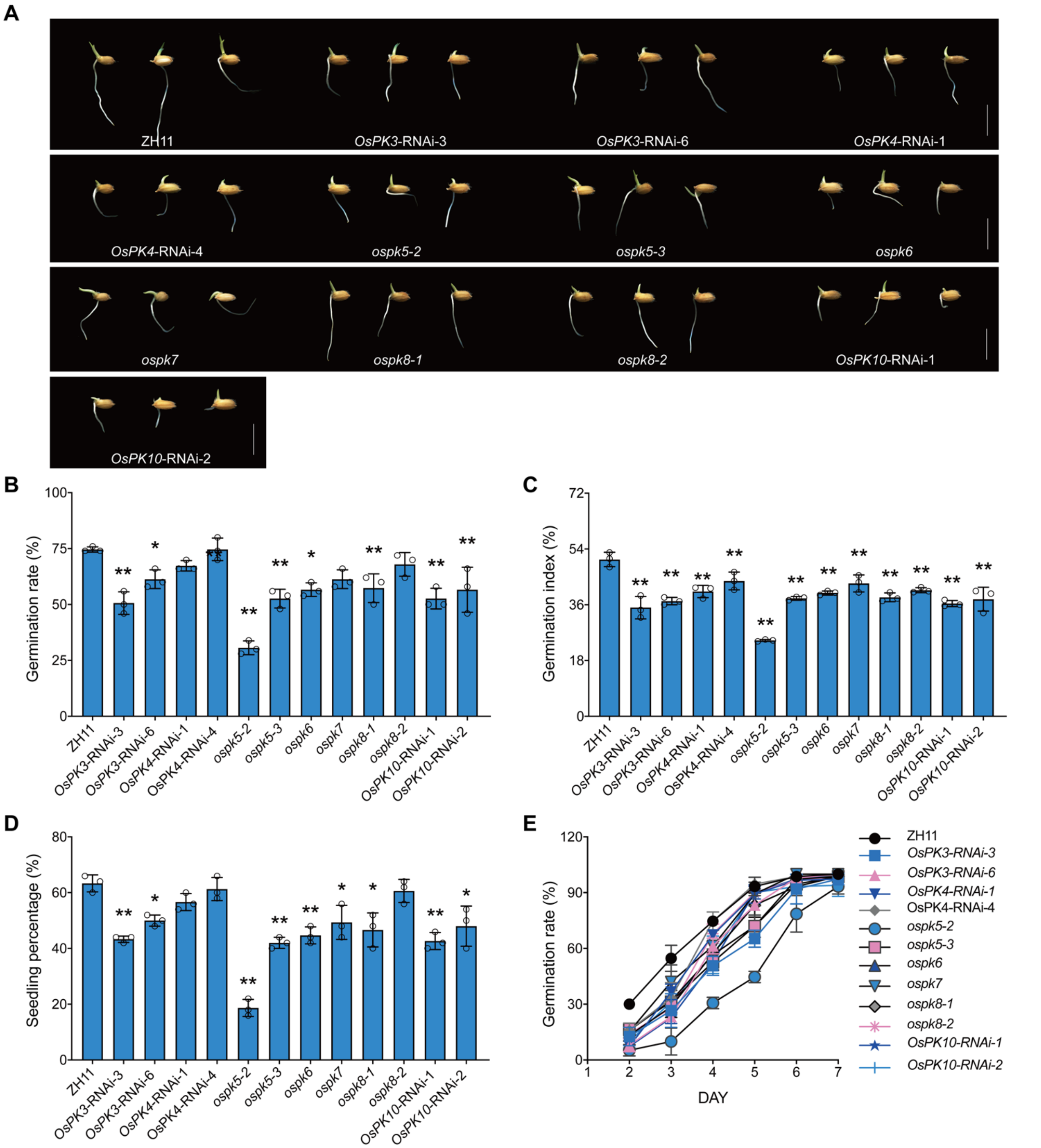

Figure S5. Seed germination of mutant and RNAi material of PK genes in rice. **(A)** Seed germination of CRISPR mutants, RNAi lines, and WT after 4 days of imbibition. Comparison of dynamic seed germination phenotype **(B)**, germination rates **(C)**, germination indexes, **(D)** and seedling percentages **(E)** between mutants, RNAi lines, and WT at 4 days after imbibition. Error bars indicate the mean ± SD of three individual replicates. Asterisks indicate statistical significance between the WT and the mutant and RNAi material seeds, as determined by a Student's *t*-test (*$P<0.05$; **$P<0.01$). Bars = 1 cm. DAY represents days after imbibition.

Table S1. Identification, protein physical and biochemical characteristics analysis of PKs in rice

Name	Locus Name	Genomic length(bp)	Length of CDS (bp)	Amino acids (aa)	Molecular Weight (KD)	Isoelectric Point	GRAVY	Instability index	Aliphatic index
OsPK1	LOC_Os11g05110	4395	1584	527	57.32	6.3	0.025	30.9	100.09
OsPK2	LOC_Os07g08340	4639	1737	578	62.87	5.92	-0.09	44.06	91.35
OsPK3	LOC_Os04g58110	3934	1536	511	55.23	8.25	0.032	31.61	99.78
OsPK4	LOC_Os12g05110	4515	1584	528	57.44	6.3	0.004	29.58	100.06
OsPK5	LOC_Os01g16960	5791	1533	510	54.905	8.14	0.047	33.3	94.31
OsPK6	LOC_Os01g47080	7340	1752	583	63.25	6.01	-0.143	38.33	87.14
OsPK7	LOC_Os03g20880	3479	1347	448	48.66	9.19	-0.194	48.51	86.67
OsPK8	LOC_Os03g46910	4097	1647	548	59.68	6.08	-0.065	35.29	93.5
OsPK9	LOC_Os10g42100	5870	1713	570	61.65	6.01	-0.159	42.13	87.12
OsPK10	LOC_Os11g10980	6539	1584	527	57.17	5.91	-0.082	35.7	92.66

Table S2. Primer used in this study

Primer name	Sequence
OsPK1-RT-F	ATTGCTGCCCATCAACTTCTC
OsPK1-RT-R	CAGCCAGAGTAGCCGAATTT
OsPK2-RT-F	CATGAGGAACTGGAACTTAAA
OsPK2-RT-R	GACGAAAACGGCATCTAC
OsPK3-RT-F	GCTCTGTCGTTTGTCCGTAA
OsPK3-RT-R	TTTCAACCTTTGACATCAGCTT
OsPK4-RT-F	GAGGCCTCCTCACAAGTGC
OsPK4-RT-R	CACCTGGCTTCACAGCCTTA
OsPK5-RT-F	GTCATGCTCGACACCAAGG
OsPK5- RT-R	TGACCCTTCTTCAACTGTACG
OsPK6-RT-F	TGTTCGTGGAAAGAGCG
OsPK6-RT-R	AAAGAAACGGCATAGTAGTCA
OsPK8-RT-F	TCCTCGAGCCAAACTGTCAT
OsPK8-RT-R	GAGCGATGCAATCAACTCCT
OsPK9-RT-F	GCAGTTCGTGAAGGTTCTGA
OsPK9-RT-R	GGATTCTGTTCTCTGTGCCA
OsPK10-RT-F	GGGCTCTGATCCAACAAAGT
OsPK10-RT-R	CTCCTCAGTACTCGCCATCA
OsPK1-1132-F	CGGGCTGCAGGAATTCATGCATTCGACGAATCTGCTGC

OsPK1-1132-R	CGGTATCGATAAGCTTATCGTCCAGCTCAATGATCTTC
OsPK2-1132-F	CGGGCTGCAGGAATTCATGGCCGCCACCGCCGCCGCGG
OsPK2-1132-R	CGGTATCGATAAGCTTAGGTACGTTCATGACCTGGATG
OsPK3-1132-F	CGGGCTGCAGGAATTCATGGCGAACATCGACATGGGG
OsPK3-1132-R	CGGTATCGATAAGCTTCTTCACGATGCAGATCTTGAT
OsPK4-1132-F	CGGGCTGCAGGAATTCATGCATTCGACGAATCTGCTG
OsPK4-1132-R	CGGTATCGATAAGCTTATCGTCCAGCTCAATGATCTTC
OsPK5-1132-F	CGGGCTGCAGGAATTCATGGCGGTGATGGAGGAGCAGC
OsPK5- 1132-R	CGGTATCGATAAGCTTGTTCGCTGTCAGGAGCTTGAT
OsPK6-1132-F	CGGGCTGCAGGAATTCATGGCGCAGGTGGTGGCTGCGG
OsPK6-1132-R	CGGTATCGATAAGCTTAACCTTCCTGACCTGAATATTA
OsPK7-1132-F	CGGGCTGCAGGAATTCATGGCCGCCGCGGTCAGGCCG
OsPK7-1132-R	CGGTATCGATAAGCTTAATGCCTTTGGTGTAGCGGTG
OsPK8-1132-F	CGGGCTGCAGGAATTCATGGCAACCTCCGCCGCCGCCG
OsPK8-1132-R	CGGTATCGATAAGCTTGTCCACTATTCGAACTTGAAT
OsPK9-1132-F	CGGGCTGCAGGAATTCATGGCGGCGGCGGCGGCTGAG
OsPK9-1132-R	CGGTATCGATAAGCTTGCCCTGGACTTTCCTCACTTGA
OsPK10-1132-F	CGGGCTGCAGGAATTCATGCAGGGCGCCAACATGCTC
OsPK10-1132-R	CGGTATCGATAAGCTTCGAACTGTCACACTCGATGAT
OsPK1-AD-F	GGAGGCCAGTGAATTCATGCATTCGACGAATCTGCTGC
OsPK1-AD-R	CGAGCTCGATGGATCCCTAATCGTCCAGCTCAATGATCTTC
OsPK2-AD-F	GGAGGCCAGTGAATTCATGGCCGCCACCGCCGCCGCGG

OsPK2-AD-R	CGAGCTCGATGGATCCTCAAGGTACGTTCATGACCTGGATG
OsPK3-AD-F	GGAGGCCAGTGAATTCATGGCGAACATCGACATGGGG
OsPK3-AD-R	CGAGCTCGATGGATCCTTACTTCACGATGCAGATCTTGAT
OsPK4-AD-F	GGAGGCCAGTGAATTCATGCATTCGACGAATCTGCTG
OsPK4-AD-R	CGAGCTCGATGGATCCCTAATCGTCCAGCTCAATGATCTTC
OsPK5-AD-F	GGAGGCCAGTGAATTCATGGCGGTGATGGAGGAGCAGC
OsPK5- AD-R	CGAGCTCGATGGATCCCTAGTTCGCTGTCAGGAGCTTGAT
OsPK6-AD-F	GGAGGCCAGTGAATTCATGGCGCAGGTGGTGGCTGCGG
OsPK6-AD-R	CGAGCTCGATGGATCCTCAAACCTTCCTGACCTGAATATTA
OsPK8-AD-F	GGAGGCCAGTGAATTCATGGCCGCCGCGGTCAGGCCG
OsPK8-AD-R	CGAGCTCGATGGATCCTCAAATGCCTTTGGTGTAGCGGTG
OsPK9-AD-F	GGAGGCCAGTGAATTCATGGCGGCGGCGGCGGCTGAG
OsPK9-AD-R	CGAGCTCGATGGATCCTTAGCCCTGGACTTTCCTCACTTGA
OsPK10-AD-F	GGAGGCCAGTGAATTCATGCAGGGCGCCAACATGCTC
OsPK10-AD-R	CGAGCTCGATGGATCCTTACGAACTGTCACACTCGATGAT
OsPK1-BD-F	CATGGAGGCCGAATTCATGCATTCGACGAATCTGCTGC
OsPK1-BD-R	TAGTTATGCGGCCGCTGCAGCTAATCGTCCAGCTCAATGATCTTC
OsPK2-BD-F	CATGGAGGCCGAATTCATGGCCGCCACCGCCGCCGCGG
OsPK2-BD-R	TAGTTATGCGGCCGCTGCAGTCAAGGTACGTTCATGACCTGGATG
OsPK3-BD-F	CATGGAGGCCGAATTCATGGCGAACATCGACATGGGG
OsPK3-BD-R	TAGTTATGCGGCCGCTGCAGTTACTTCACGATGCAGATCTTGAT

OsPK4-BD-F	CATGGAGGCCGAATTCATGCATTCGACGAATCTGCTG
OsPK4-BD-R	TAGTTATGCGGCCGCTGCAGCTAATCGTCCAGCTCAATGATCTTC
OsPK5-BD-F	CATGGAGGCCGAATTCATGGCGGTGATGGAGGAGCAGC
OsPK5- BD-R	TAGTTATGCGGCCGCTGCAGCTAGTTCGCTGTCAGGAGCTTGAT
OsPK6-BD-F	CATGGAGGCCGAATTCATGGCGCAGGTGGTGGCTGCGG
OsPK6-BD-R	TAGTTATGCGGCCGCTGCAGTCAAACCTTCCTGACCTGAATATTA
OsPK8-BD-F	CATGGAGGCCGAATTCATGGCCGCCGCGGTCAGGCCG
OsPK8-BD-R	TAGTTATGCGGCCGCTGCAGTCAAATGCCTTTGGTGTAGCGGTG
OsPK9-BD-F	CATGGAGGCCGAATTCATGGCGGCGGCGGCGGCTGAG
OsPK9-BD-R	TAGTTATGCGGCCGCTGCAGTTAGCCCTGGACTTTCCTCACTTGA
OsPK10-BD-F	CATGGAGGCCGAATTCATGCAGGGCGCCAACATGCTC
OsPK10-BD-R	TAGTTATGCGGCCGCTGCAGTTACGAACTGTCACACTCGATGAT
OsPK3Ri-K-F	TTACTTCTGCACTAGGTACC GTACGCACAGCCAACAAGG
OsPK3Ri-S-R	CGTAGGGGCGATAGAGCTCGCAGAGCAACAACAGCATCA
OsPK3Ri-P-F	TGCTTTTGGTTTTCTGCAGGCAGAGCAACAACAGCATCA
OsPK3Ri-B-R	GAATTCCCGGGGATCC GTACGCACAGCCAACAAGG
OsPK4Ri-K-F	TACTTCTGCACTAGGTACCCCATCTCCTCCTGCCTCAAG
OsPK4Ri-S-R	TAGGGGCGATAGAGCTCACACAGACCACATCATCTCCTT
OsPK4Ri-P-F	CTTTTGGTTTTCTGCAGACACAGACCACATCATCTCCTT
OsPK4Ri-B-R	GAATTCCCGGGGATCCCCATCTCCTCCTGCCTCAAG
OsPK10Ri-K-F	TTACTTCTGCACTAGGTACCTTGCTTGGTGCGGAGACT
OsPK10Ri-S-R	CGTAGGGGCGATAGAGCTCATTGCCTTGCCTGAGTTGTG
OsPK10Ri-P-F	TGCTTTTGGTTTTCTGCAGATTGCCTTGCCTGAGTTGTG
OsPK10Ri-B-R	GAATTCCCGGGGATCCTTGCTTGGTGCGGAGACT
